# Three-dimensional topology dataset of folded radar stratigraphy in northern Greenland

**DOI:** 10.1038/s41597-023-02339-0

**Published:** 2023-08-07

**Authors:** Steven Franke, Paul D. Bons, Kyra Streng, Felicitas Mundel, Tobias Binder, Ilka Weikusat, Catherine C. Bauer, John D. Paden, Nils Dörr, Veit Helm, Daniel Steinhage, Olaf Eisen, Daniela Jansen

**Affiliations:** 1 Department of Geosciences, Tübingen University, Tübingen, Germany; 2 Alfred Wegener Institute, Helmholtz Centre for Polar and Marine Research, Bremerhaven, Germany; 3 School of Earth Science and Resources, China University of Geosciences, Beijing, China; 4 Center for Remote Sensing and Integrated Systems (CReSIS), University of Kansas, Lawrence, KS USA; 5 Karlsruhe Institute of Technology, Karlsruhe, Germany; 6 Department of Geosciences, University of Bremen, Bremen, Germany

**Keywords:** Cryospheric science, Geodynamics

## Abstract

We present a dataset of reconstructed three-dimensional (3D) englacial stratigraphic horizons in northern Greenland. The data cover four different regions representing key ice-dynamic settings in Greenland: (i) the onset of Petermann Glacier, (ii) a region upstream of the 79° North Glacier (Nioghalvfjerdsbræ), near the northern Greenland ice divide, (iii) the onset of the Northeast Greenland Ice Stream (NEGIS) and (iv) a 700 km wide region extending across the central ice divide over the entire northern part of central Greenland. In this paper, we promote the advantages of a 3D perspective of deformed englacial stratigraphy and explain how 3D horizons provide an improved basis for interpreting and reconstructing the ice-dynamic history. The 3D horizons are provided in various formats to allow a wide range of applications and reproducibility of results.

## Background & Summary

Over the last decades, the Arctic, and thus, the Greenland ice sheet (GrIS), has warmed more intensively than other regions^1,2^. The resulting trend in mass loss of the GrIS contributes to sea-level rise of approximately 0.8 mm/a^3^. Approximately half of the mass loss is attributed to the ice-dynamical contribution from the acceleration of GrIS’ marine-terminating glaciers^4,5^. However, in contrast to the ice-mass loss driven by melting, the projected contributions to sea-level rise due to ice dynamics are associated with high uncertainties^6^. Over the last decade, critical regions in the GrIS experienced extensive speedup and thinning due to frontal ice retreat, affecting the inland ice-flow dynamics^7^. To better predict future dynamic mass losses, studying past ice-dynamical processes can provide important insight and constraints on forward ice-flow modelling.

The GrIS has been extensively mapped with radio-echo sounding (RES) surveys to quantify the thickness of the ice and to map the englacial stratigraphy^8–11^. The transmitted electromagnetic waves from the RES system get reflected at interfaces of dielectric contrasts^12^ within the ice column at so-called internal reflection horizons (IRHs). In the shallow and middle section of the ice column, IRHs primarily represent paleo surfaces that are caused by the former deposition of volcanic material^13^ at the surface and subsequently buried by subsequent snow accumulation, and hence, represent a horizon of a consistent time of deposition^14^. These IRHs provide a detailed insight into the englacial stratigraphy^8,15–18^.

Viewing the englacial stratigraphy of IRHs at high spatial resolution and in three dimensions has spatial advantages, potentially allowing better process understanding within and at the boundaries of the ice sheet, such as deformation, accumulation, melting and freezing. Three-dimensional views of IRHs within the ice sheets are obtained by tracing IRHs along RES profiles and then interpolating surfaces of equal age between different profiles, allowing the reconstruction of the 3D character of IRHs^19^ (which we will refer to as 3D horizons). This way, many of the processes occurring in the past, such as changes in the flow and melting of ice sheets, are preserved in the ice and can be decoded^20–25^.

This paper describes the publication of 3D horizons in the Greenland ice sheet. The horizons reflect the deformation history in different ice-dynamic regimes. A previous study has shown that 3D horizons can provide a holistic overview of the spatial variations in the character of the ice, such as deciphering folding processes due to the mechanical anisotropy of ice at the onset of ice streams^19^. Furthermore, 3D horizons revealed the past activity of paleo-ice streams in currently slow-flowing regions in northern central Greenland^21^. In addition to the publication of the data in various file formats, we explain in detail how and on what basis the 3D horizons were generated. The datasets are archived and available in a Pangaea Publication Series: 10.1594/PANGAEA.954991^26^.

## Methods

### Study regions

**Fig. 1:**
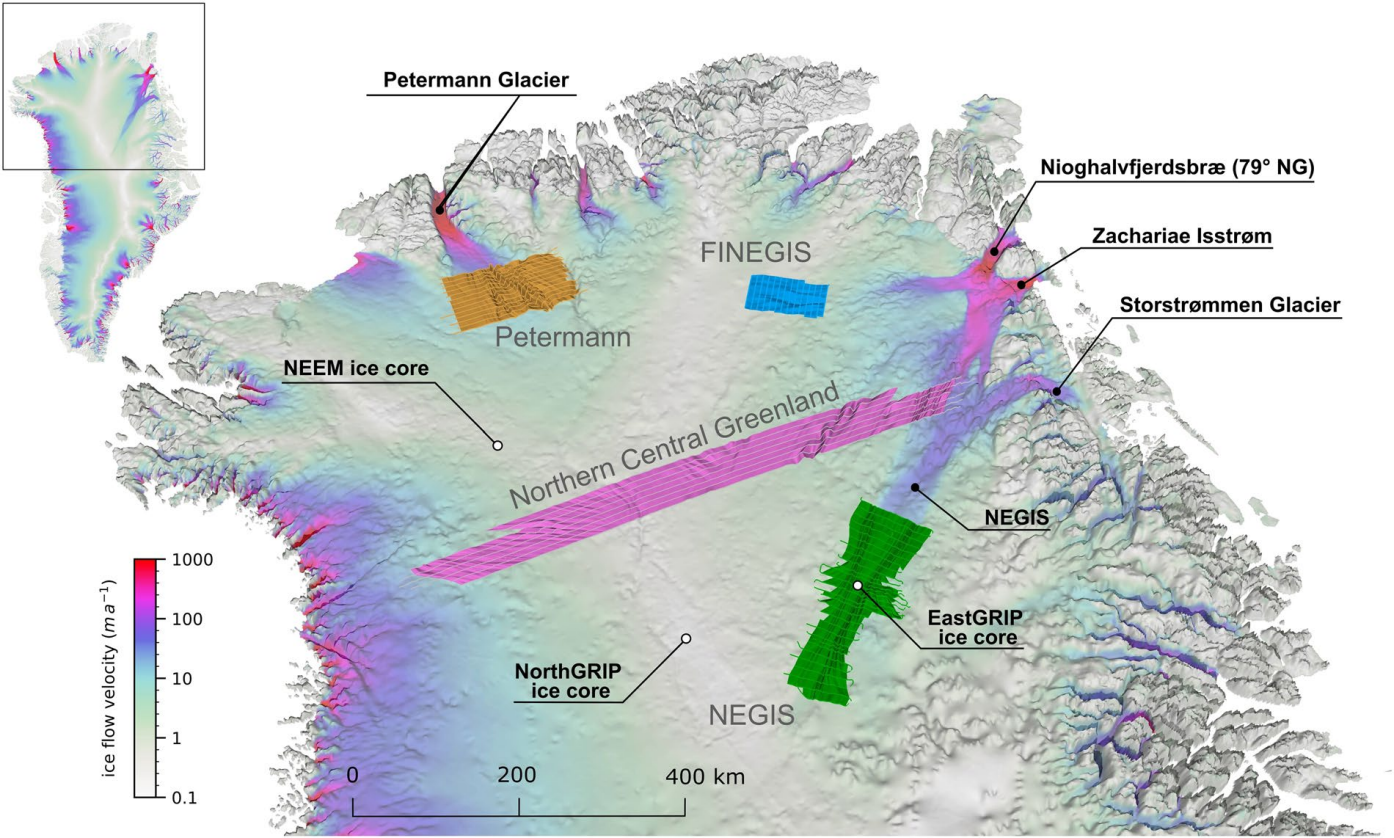
Overview of the locations of the four data sets of 3D englacial stratigraphy horizons in northern Greenland: Petermann (orange), FINEGIS (Folds in the Northeast Greenland ice sheet; blue), NEGIS (Northeast Greenland Ice Stream onset; green) and Northern Central Greenland (violet). The base map shows the bed topography^47^ overlain with a colour map of the ice surface velocity^85^ of Greenland. The map has a vertical exaggeration of 15, and the ice surface velocity is shown on a logarithmic scale.

The data presented in this study originate from four different regions in northern Greenland (Fig. 1) that represent different regional glaciological and ice-dynamic settings: (i) the onset region of fast ice flow of the Petermann Glacier (Petermann Gletsjer), (ii) a region upstream of the 79° North Glacier (Nioghalvfjerdsbræ) in the vicinity of the ice divide (FINEGIS; Folds in the northeast Greenland ice sheet), (iii) the upstream region of the Northeast Greenland Ice Stream (NEGIS), and (iv) a large area covering Northern Central Greenland from the west over the central ice divide to the east.

#### Petermann glacier

The first data set presented here covers the onset of the Petermann Glacier (PG). PG is located in northwest Greenland and represents one of Greenland’s largest outlet glaciers, draining about 4% of the GrIS^27^. The ice shelf of the PG is confined by a fjord and represents the longest floating tongue in Greenland^28^. Recent calving events in 2010 and 2012^29^ have substantially shortened the ice shelf, which caused an average ice-flow acceleration of ∼10% between 2012 and 2017^30^. Further concerns have been raised that subsequent future ice shelf loss might lead to speedup of the grounded part of PG, leading to irreversible grounding line retreat and accelerated mass loss^30–33^.

**Fig. 2:**
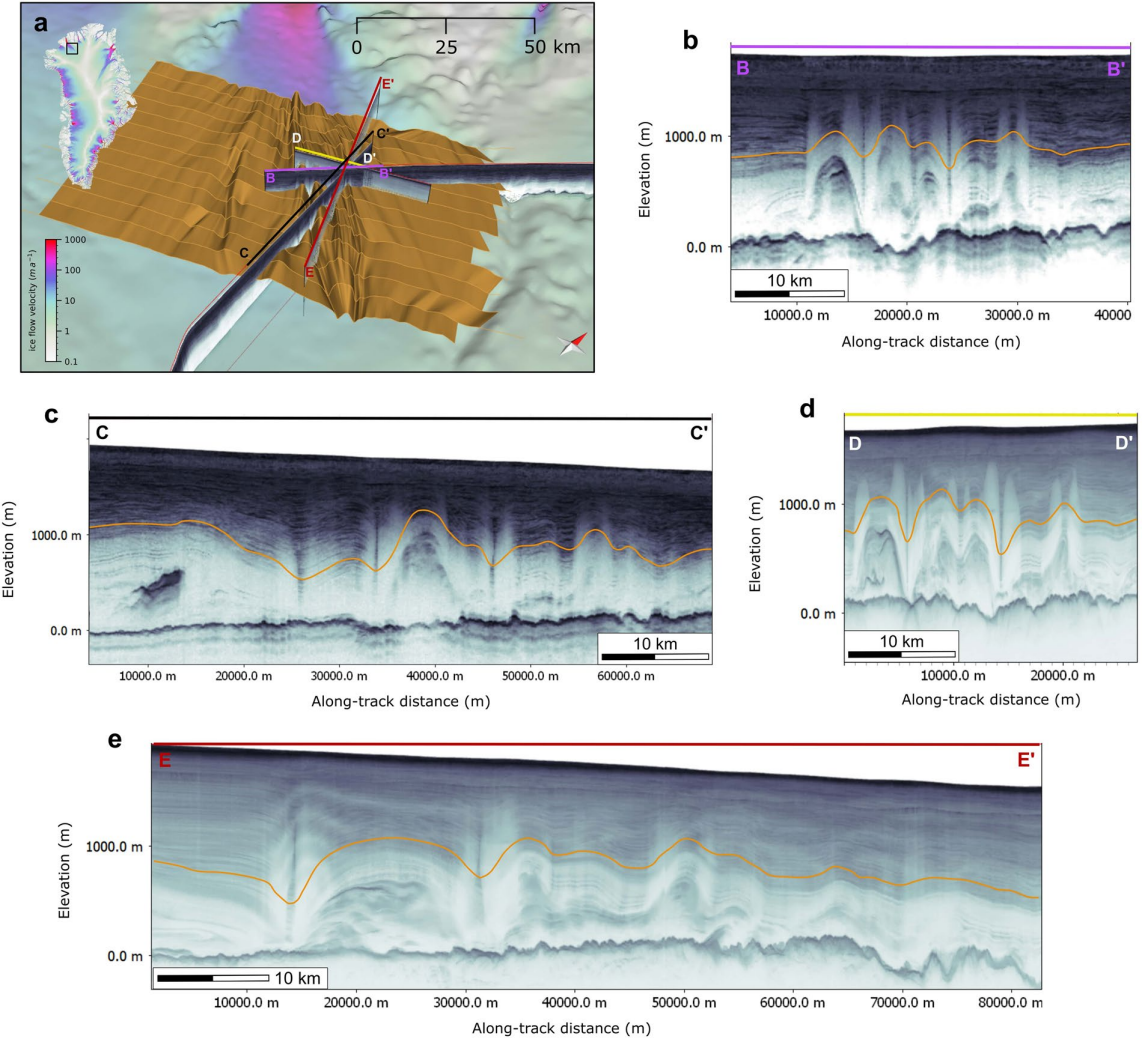
Folded IRH observed from different perspectives in RES data. (**a**) Overview map showing the P1 Petermann horizon and four RES profiles at different orientations (**b**–**e**). The y-axis (Elevation) of the radargrams is relative to the WGS84 ellipsoid, and the orange line in the radargrams (**b**-**e**) represents the IRH of the P1 3D horizon schematically.

The upstream regions of fast flow are not topographically confined by a trough^34^. The basal conditions at the onset of the PG show a complex thermal transition at the base near the onset of fast ice flow^35^. Moreover, PG’s fast flow onset region is associated with folded and discontinuous IRHs buried deep in the ice sheet^19^, ^36–38^. The folding of the stratigraphy at PG’s onset has been attributed to various processes: Several studies point to basal conditions in the form of moving patches of subglacial slip^39^ and basal freeze-on^36^. By contrast, Bons *et al*.^19^ used the complete and extensive grid of NASA’s Operation IceBridge (OIB) RES profiles^11^ to construct 3D horizons, thus overcoming the limitations of using the interpretation on individual RES profiles only, as done in the previous two studies^36,39^. For the first time, this revealed the entire 3D geometry and orientation of the englacial folds (Fig. 2). Using the orientation of the fold axes, the authors concluded that converging flow and the mechanical anisotropy of the ice sufficiently explained the large-scale folding at the onset of the PG and no spatial variations in physical properties at the base need to be taken into account.

#### Folds in the northeast greenland ice sheet (FINEGIS)

The second data set is located in northeast Greenland in the upstream part of the northern catchment of the 79° North Glacier. The region extends approximately from 79°N to 80°N and from 32°W to 40°W (FINEGIS in Fig. 1). Ice surface velocity in this region is almost zero in the western part as it is located close to Greenland’s central ice divide. Further east, ice flow velocity increases up to 15 ma^−1^. Two cylindrical fold units in this area are partly intersected by OIB RES profiles and have been subject to prior studies^37,38,40^. A systematic structural analysis of these folds based on additional high-resolution RES data allowed the creation of 3D horizons of the folded englacial stratigraphy. The geometry and deformation patterns of the folds were attributed to the time-varying activity of a now-extinct ice stream that first changed the flow pattern in its catchment and then was deactivated in the Holocene^21^. Locally this ancient ice-flow regime must have been much more focused and reached further inland than today.

#### Northeast Greenland Ice Stream onset region

The third data set covers the upstream part of the Northeast Greenland Ice Stream (NEGIS). Its onset region is close to the central divide, more than 500 km inland from its outlets (Fig. 1). In the course of predicting the future behaviour of the GrIS, the NEGIS represents one of the largest uncertainties for ice flow predictions^41^. Near its outlets^42^ (Zachariæ Isstrøm and Storstrømmen Glacier; Fig. 1), NEGIS is increasingly losing mass over the last decades^43^, mainly due to the retreat of the grounding line of NEGIS’ outlet glaciers (e.g., the rapid retreat of Zachariae Isstrom^44^). The resulting thinning and flow acceleration has the potential to propagate upstream^45^. Acceleration rates in the order of a few cma^−1^ at EastGRIP (East Greenland Ice-core Project)^7^,^46^ are first indications that the whole of NEGIS may not be in equilibrium.

Apart from its exceptional length in the Greenlandic context, NEGIS is also unique regarding its distinct and continuous shear margins along its entire length. The position of these shear margins appears not to be constrained by the underlying bed topography^47,48^. However, the geometry of the ice stream, the position of its shear margins, and the origin of NEGIS itself are still not fully understood^21^,^49–54^. Progress in this field is expected from analyzing the EastGRIP ice core^55,56^, GPS measurements^46,57^, an extensive grid of RES profiles flown over NEGIS^58,59^, ground-based phase sensitive RES (pRES) measurements^60,61^, and modelling^62–64^.

#### Northern central greenland

The fourth data set covers a ∼700 km wide strip extending across the central ice divide over the entire northern part of central Greenland (Fig. 1). In the east, the data set extends into the shear margin of NEGIS at its downstream end; in the west, into the region of elevated flow velocity of the northwest Greenland outlet glaciers. Characteristic features that the dataset covers in the central area and that define the local radio-stratigraphy are, for example, the englacial imprint in the stratigraphy of a paleofluvial mega-canyon in the bed topography^65^ and numerous plume-like folds, which were attributed to basal freeze-on processes^38^.

### The perspective of 3D englacial stratigraphy

The way the geometry of IRHs imaged in radargrams is perceived depends on the orientation of the RES profile in relation to the three-dimensional geometry of the englacial stratigraphy — in short, the cutting angle. For example, cylindrical folds, such as those at Petermann Glacier^19^, appear very different depending on the orientation of the RES profile (Fig. 2). The geometries of the folds change depending on the angle of incision and appear misleading if viewed at angles other than 90° to the orientation of the fold axis (Fig. 2b,d). The greater the deviation from this orientation, the longer the wavelength of these folds appears (Fig. 2c,e). These distorted geometries can lead to significant misunderstandings and misinterpretations of processes involved in the formation of folds. Three-dimensional reconstruction provides remedy. We, therefore, consider it essential, especially for understanding the formation processes over time and spatial classification of deformations of the englacial stratigraphy^19,21^.

### Workflow

**Fig. 3:**
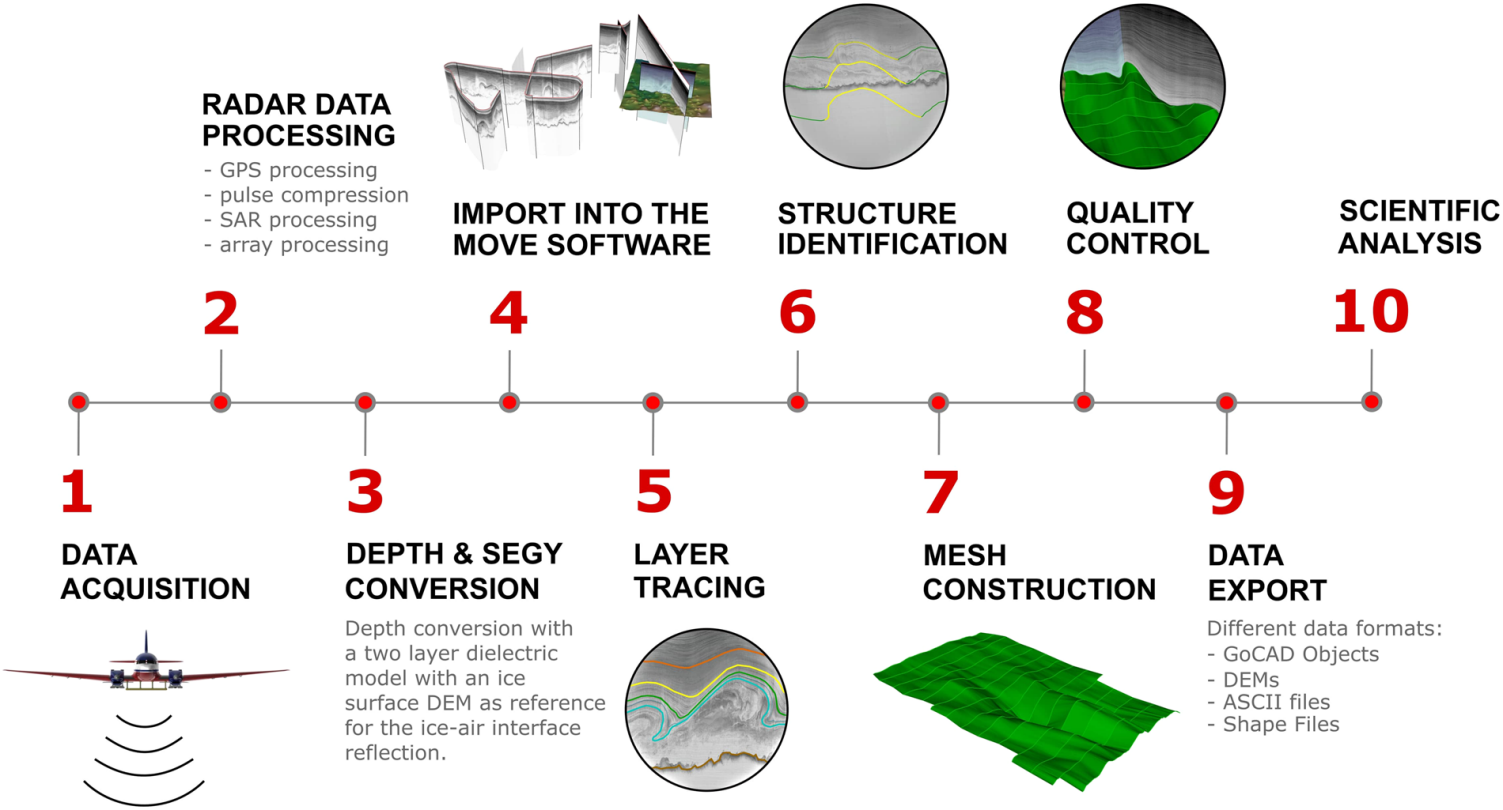
Workflow sequence for 3D horizon construction from RES data acquisition to the scientific analysis of 3D horizons.

The construction of 3D horizons begins with RES data acquisition over the ice sheet. Several steps are necessary before the full 3D product is produced, which are presented in this section. A schematic overview of the steps is shown in Fig. 3.

#### Data sources and data acquisition

The basis for generating the data products published here is airborne RES data. The RES data used to create the 3D horizons presented here were acquired with multi-channel coherent depths sounders (MCoRDS) flown by the Center for Remote Sensing and Integrated Systems (CReSIS) and NASA’s Operation IceBridge^11,66^ as well as surveys from the Alfred Wegener Institute, Helmholtz Centre for Polar and Marine Research (AWI)^21,58,67^ with AWI’s polar research aircrafts^68^. The radar specifications for the RES data used in each of the four survey regions are shown in Table 1. A complete list with all RES profiles used for the reconstruction of the 3D horizons is shown in Table 2.

**Table 1:** RES data system specifications and acquisition parameters of the data used for 3D horizon construction.

Region	Scientific reference	Platform	RES System	Frequency Range (MHz)	Number of transmitters	Year
Petermann Glacier	Bons *et al*.^19^	NASA DC-8	MCoRDS	189.15–198.65	8	2010
		NASA P-3B	MCoRDS 2	180–210	16	2011
FINEGIS	Franke *et al*.^21^	AWI Polar6	MCoRDS 5	180–210	8	2018
NEGIS onset	Franke *et al*.^58^	AWI Polar6	MCoRDS 5	180–210	8	2018
NC Greenland	None	NASA P-3	MCoRDS 2	180–210	16	2012
		NASA P-3	MCoRDS 3	180–210	16	2013
		NASA P-3	MCoRDS 3	180–210	16	2014

**Table 2:** RES profiles used to create the 3D horizons of the four data sets.

Data Set	Campaign	Segment	Frames
Petermann Glacier	2010 Greenland DC8 (NASA OIB)	20100324_01	011–034
	2011 Greenland P3 (NASA OIB)	20110429_01	009–034
		20110429_02	001–009
		20110507_01	011–038
		20110507_02	001–004
			017–020
FINEGIS	2018 Greenland Polar6 (AWI)	20180414_09	002–010
		20180415_06	001–007
		20180418_03	006–014
NEGIS onset	2018 Greenland Polar6 (AWI)	20180508_02	002
		20180509_01	007–013
		20180510_01	005–007
		20180511_01	005–011
		20180512_01	010–012
		20180512_02	007–009
		20180514_01	009–017
		20180514_03	005–009
		20180517_01	008–018
NC Greenland	2012 Greenland P3 (NASA OIB)	20120508_04	001–018
		20120508_05	001–006
		20120508_06	008–016
		20120508_07	001–011
		20120507_03	008–012
		20120507_04	001–002
		20120507_05	001–014
		20120507_06	030–033
		20120507_07	001–015
	2013 Greenland P3 (NASA OIB)	20130419_01	012–024
			047–060
	2014 Greenland P3 (NASA OIB)	20140521_02	011–021
			052–061

#### Radio-echo sounding data processing

The RES data processing for all MCoRDS systems was performed with the CReSIS Toolbox^69^ and consisted of four main steps: (1) GPS synchronization, (2) pulse compression of the chirped waveform in the vertical range to improve the range signal quality and the signal-to-noise ratio (SNR), (3) Along-track Synthetic Aperture Radar (SAR) processing (frequency-wavenumber migration), and (4) cross-track (array) processing to achieve a coherent combination of the return signals of the antenna array to increase SNR and reduce surface clutter^66^. For detailed acquisition and processing settings of AWI’s 2018 Greenland Polar6 campaign, see Franke *et al*.^48,58^. For acquisition and processing details of NASA’s OIB campaigns, we refer to the CReSIS Open Polar Server^70^ and the CReSIS RDS (radar depth sounder) documentation^71^.

We used the SAR-processed RES product as data basis for IRH tracing for the subsequent construction of the 3D horizons. We converted the RES data from the two-way travel time (TWT) domain into the elevation domain. For the conversion from TWT to elevation, we used a two-dimensional dielectric constant (ε) model for air with ε_air_ = 1 and for ice with ε_ice_ = 3.15^72^. To attribute the absolute elevation to the RES data, we linked the ice surface elevation from the Greenland Ice Mapping Project (GIMP)^73^ with the location of the ice surface reflection in the RES data. Thus, all elevations are in height above the WGS84 ellipsoid^73^. We defined suitable IRHs as those that were easily recognizable in all radargrams and had a drill-core constrained age relevant to the respective research questions.

#### Import into the *Move* software and IRH tracing

For the NEGIS, FINEGIS and NC Greenland data sets, elevation-converted RES data were exported to the SEGY format with the ObsPy Python framework^74^. The radargrams in the SEGY format were imported into the 3D structural modelling software *Move*. A slightly modified workflow was used for the Petermann data set. The elevation-converted radargram plots (jpeg files) from CReSIS^75^ for the respective RES profiles in this region were cropped and provided with a corresponding coordinate system. Similarly to the SEGY format, these radargram plots with coordinates were imported into the *Move* 3D canvas. Compared to converting the RES data into the SEGY format, this method does not provide the full resolution of the radar data and also has slight inaccuracies with the correct representation of the IRHs in 3D space. For example, small turns in the flight trajectory will be displayed as straight in the radargram plots. However, it is sufficiently accurate for mapping the geometries of IRHs in the frame of the research question for the study by Bons *et al*.^19^.

After the RES sections for a given region were imported into *Move*, they were inspected for the predominant deformation patterns. For the subsequent construction of the 3D horizons, the RES segments and thus the orientation of the IRHs to be traced must be oriented at a high angle to the fold structures where possible (i.e., perpendicular to the orientation of the fold axis). All IRHs were traced manually in *Move* without an auto picker.

#### Generation of 3D horizons

**Fig. 4:**
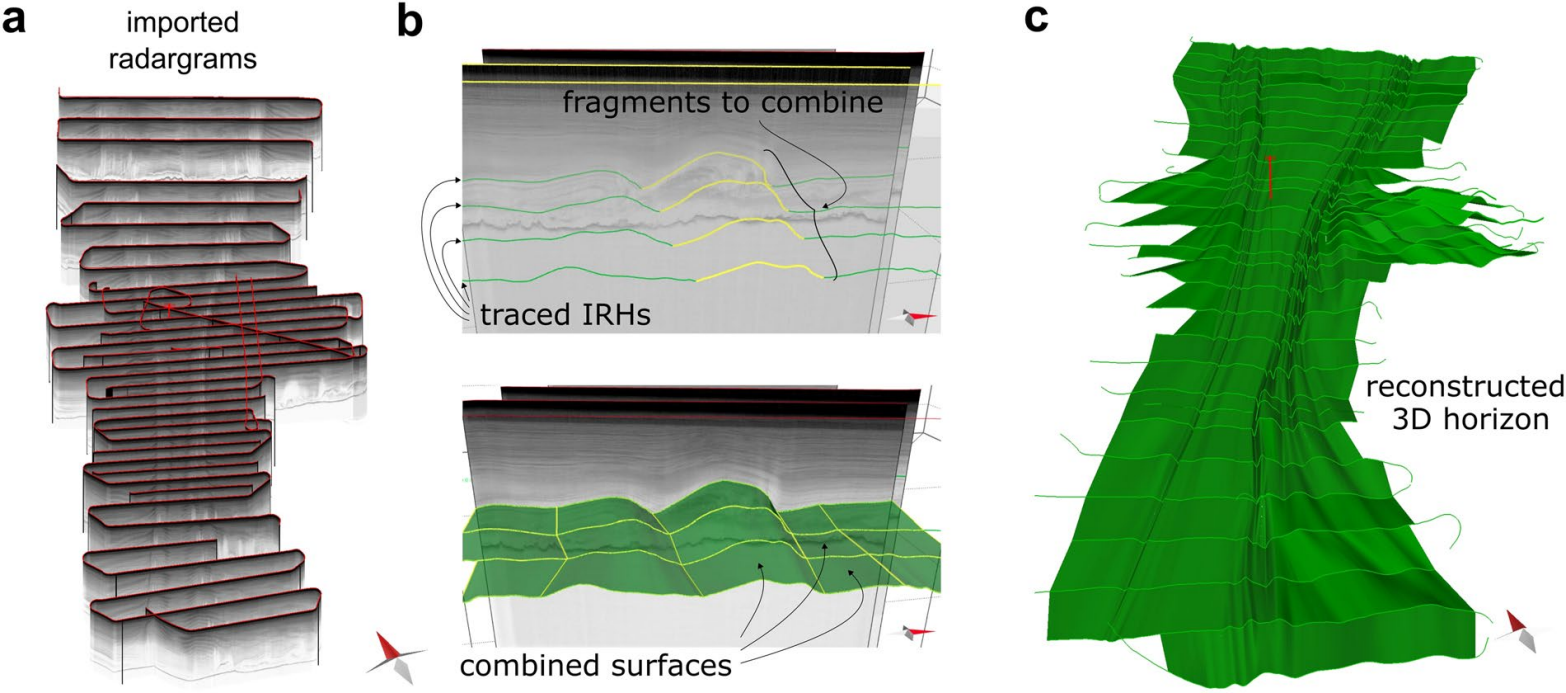
Example of the reconstruction of a 3D horizon from traced IRHs: (**a**) radargram import, (**b**) IRH tracing and fragment combination, and (**c**) a large 3D horizon composed of many combined surfaces.

The workflow of a 3D horizon construction based on traced IRHs is shown in Fig. 4. The IRHs were subdivided into shorter line segments that clearly define the same structure in the neighbouring IRHs (e.g., an anticline in the upper panel of Fig. 4b). A horizon was then fitted through these sections using linear interpolation. All these steps are performed in the *Move* software. A complete 3D horizon is thus composed of several small single 3D horizons (lower panel in Fig. 4b). The quality of the 3D horizon reconstruction depends on the spacing of the RES profiles relative to the length scale of structures, such as folds. This means there is a certain user-dependent uncertainty in terms of the choice of structures that are connected when they vary strongly from one RES profile to the next.

#### 3D horizon age attribution

For the age attribution of the 3D horizons, we traced the corresponding IRHs to one of the nearby deep ice core sites (Fig. 1) and assigned the ice core’s age to the corresponding depth of the IRH at the ice core location. For the NEGIS horizon age attribution, we used the RES profile 20180508_06_004 from the EGRIP-NOR-2018 campaign^58^ and the EGRIP GICC05-EGRIP-1 timescale^76^. The closest distance between the RES profile and the ice core location is 5 m. The ages of the FINEGIS, Petermann Glacier and NC Greenland data sets were determined based on the OIB RES profile 20110506_02_008 in combination with the NEEM ice core chronology (GICC05modelext-NEEM-1 timescale)^77^. The closest distance between the OIB Profile to the NEEM ice core is ~170 m, and for the IRH tracing towards the 3D horizons, we used the following OIB RES profiles: 20110506_02_[001–008], 20170413_01_[039–043], 2013042601_[051–055], and 20170413_01_[048–055]^75^.

Using these ice-core stratigraphies, we dated the 3D horizons, which span approximately the last 7–60 ka. We traced three horizons over FINEGIS, with ages between 60 and 45.5 ka. Over Petermann and NC Greenland, we traced two horizons with ages of 12 and 37.5 ka, and 13.8 and 37.5 ka, respectively. Finally, one horizon was traced over NEGIS, with an estimated age of 7.3 ka. Further details are provided in Table 3 and in the Age validation section. The ages of the 45.5 and 52.2 ka horizons in the FINEGIS data set, as well as the 37.5 ka horizons for the Petermann and NC Greenland data sets were determined from known ages of three prominent RES reflectors, which are present in all RES profiles used here. The age of the F3 60.0 ka horizon was estimated by linear interpolation with depth, assuming that the age increases with the same function of depth as between the 45.5 and 52.2 ka horizons. The 13.8 ka horizon of the NC Greenland data set and the 12.0 ka horizon of the Petermann data set were chosen because they represent clear reflections throughout the respective data set and are close to the base of the Holocene (~11.5 ka before present). The age of the NEGIS horizon was chosen because of its good visibility in the radargrams and because it represents a time horizon approximately in the middle of the Holocene (7.3 ka). The ages of the horizons are here reported with a 2.5–7.5% uncertainty with respect to the total IRH age (see Age validation section and Table 3). However, the assigned ages may potentially change, for example, due to recalibration of drill core data or more comprehensive and precise dating methods (e.g., using dielectric profiling in combination with synthetic radar modelling^78^). Nonetheless, as the primary purpose of constructing the 3D horizons is the visualization of the geometry of the stratigraphic IRHs, this is, in most cases, not a critical issue.

**Table 3:** 3D horizon specifications of the three survey regions.

Region	Scientific reference	Horizon abbreviation	Horizon age	Surface area
Petermann Glacier	Bons *et al*.^19^	P1	12.0 ± 0.9 ka	13,762 km^2^
		P2	37.5 ± 2.1 ka	12,235 km^2^
FINEGIS	Franke *et al*.^21^	F1	45.5 ± 2.5 ka	5,402 km^2^
		F2	52.2 ± 2.7 ka	5,325 km^2^
		F3	60.0 ± 4.0 ka	4,781 km^2^
NEGIS onset	Franke *et al*.^58^	N1	7.3 ± 0.2 ka	15,451 km^2^
Northern Central Greenland		NG1	13.8 ± 1.0 ka	32,539 km^2^
		NG2	37.5 ± 2.1 ka	30,359 km^2^

The scientific reference column represents the link of the data set to a publication where either the 3D horizons were analyzed or the RES data of the corresponding survey were published.

## Data Records

The data related to this publication is available at PANGAEA (10.1594/PANGAEA.954991)^26^ and represent 3D horizon geometries constructed using IRHs on the basis of RES data. The data sets represent the geometry of a respective IRH in four regions of the GrIS. For each region, there may be several data sets that represent different time horizons of deposition on the ice surface and are thus located at different depths within the ice sheet (this implies that older horizons found at greater depths and younger horizons at shallower depths). A summary of the key data for the different 3D horizons of the four regions is shown in Table 3.

### Data formats

The 3D horizons are provided in three data formats (Table 4): (i) Point cloud data in a tabular column-separated ASCII format, (ii) rasterized digital elevation models (DEMs), and (iii) GoCAD objects. For each dataset, we provide the 3D horizons (meshes) and the traced IRHs from the radargrams (except for the DEM data, where we only provide meshes). We have chosen these three data formats to make them available to the user for different scientific applications and to be compatible with various software (Table 4). Some data formats can be used with standard and open-source software (e.g., the DEMs and the tabular point data), but others require specialized and often commercial software (e.g., GoCAD Objects files). The rasterized DEMs are not provided for horizons containing overturning structures (fold in which both the limbs dip in the same direction, e.g. the FINEGIS horizons).

**Table 4:** Data format specifications, description and usage.

Data Type	Data Format	Description	Usage
Tabular	Point Set (.dat)	Points with coordinates (x,y,z) and scalar/vector properties saved in ASCII file format for 3D horizons	Generic ASCII data format; native format for many GIS applications and geo libraries
	Line Set (.dat)	Points with coordinates (x,y,z) and scalar/vector properties saved in ASCII file format for the traced IRHs	
Digital Elevation Model (DEM)	GeoTIFF (.tif)	Raster file generated from the 3D horizon in a georeferenced TIFF file (only available for structures which are not overturning)	Native file format for scientific programming languages and GIS applications
	NetCDF (.nc)	Raster file generated from the 3D horizon in a georeferenced NetCDF file (only available for structures which are not overturning)	
Geological Object (GoCAD Object)	TSURF (.ts)	Triangulated surface objects containing vertex coordinates and triangle-to-vertex connectivities	Mostly commercial geological modelling software with a 3D canvas
	PLINE (.pl)	Lines composed of connected (or disconnected) segments representing the traced IRHs	

The following describes the different data formats and discusses their potential applications, advantages, and disadvantages. The coordinate system for data products is the cartesian EPSG:3413 (WGS 84/NSIDC Sea Ice Polar Stereographic North). All elevations are in height relative to the WGS84 ellipsoid^73^.

**Fig. 5:**
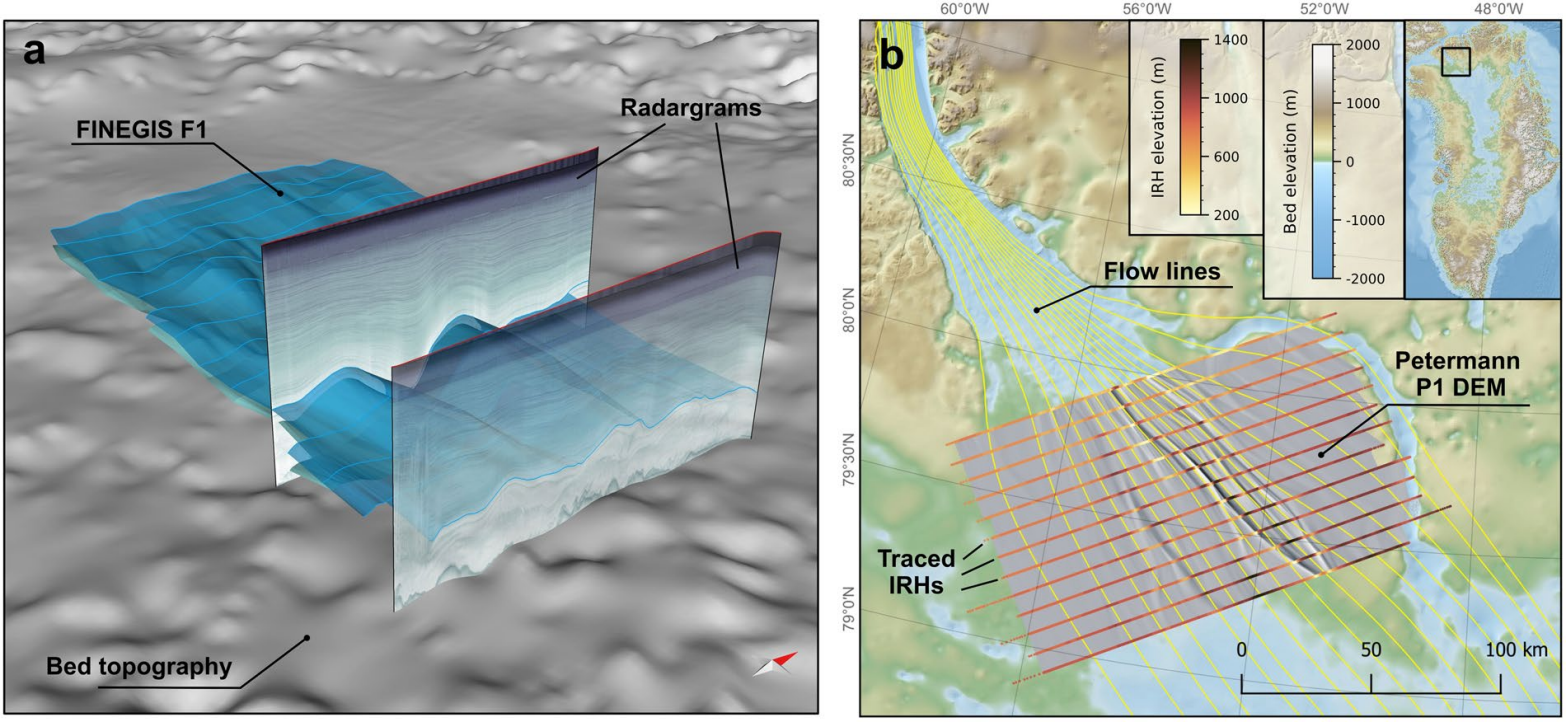
Example of the usage of different file formats. (**a**) A 3D canvas in *Move* where two of the FINEGIS GoCAD mesh objects have been imported. Furthermore, two RES sections (imported as a SEGY) and the bed topography (imported as a GeoTIFF) are shown. (**b**) The hillshaded Petermann Glacier P1 DEM is superimposed on Greenland’s bed topography^47^ in a QGIS 2D canvas. The color-coded vertical lines show the elevation of the traced isochrones (imported into QGIS from the xyz ASCII Line Set data). The yellow lines represent flow lines of the ice surface velocity^85^.

1. We provide high-resolution point clouds of xyz data in a tabular ASCII file format for all 3D horizons. The point clouds are a representation of the 3D geometry (triangulated meshes in *Move*) as single points and provided for the 3D horizons as well as for the traced IRHs. The x and y columns represent the coordinates in the EPSG:3413 coordinate system, and the z value is the elevation in meters. Distance between the points ranges from 30 to 40 meters.
2. Based on the high-resolution point cloud data, we generated digital elevation models (DEMs) in the GeoTIFF (.tif) and NetCDF (.nc) format. DEMs were interpolated to a cell size of 50 m with the SAGA GIS^79^ (version 2.2.5) Cubic Spline Approximation module^80^. The module approximates irregular 2D data in specified points using a continuous bivariate cubic spline. Here, we use 3 to 20 points locally involved in the spline calculation with five points per cell and a relative tolerance multiple in fitting spline coefficients of 140. We restrict the DEM generation to all 3D horizons that do not include overturned fold limbs (such as those in the FINEGIS data set). Although this is a considerable shortcoming when considering the complete topological analysis of those structures, the advantage of this file format is that it is a standard raster format for many GIS applications (such as QGIS and ArcGIS) and other software.
3. GoCAD (Geological Objects) is a common file format for geological modelling software. The 3D horizon meshes are stored in the triangulated surfaces (TSURF) format (.ts), which represents the shape as well as the meta-data of the respective 3D horizon. The traced IRHs are stored in the Polyline (PLINE) format (.pl), equivalent to the TSURF format, but used for 2D lines. GoCAD objects represent the 3D geometry in the highest possible resolution and have the advantage that they can be easily imported into many (mostly) commercial geology software packages with a 3D canvas and combined with other data containing geographic information, such as GeoTIFFs, shapefiles or SEGY sections (Fig. 5).

### Nomenclature of file names

The file names for the different datasets are organized in the following systematic to specify the region, horizon age, geometry and file type: “[Region]_[Horizon]_[Version]_[age]_[subset]_[geometry].[filetype]”, e.g., Petermann_P1_V001_12_0_ka_mesh.dat for the P1 12.0 ka horizon mesh point cloud in the ASCII format. An overview and explanation for the field names are shown in Table 5.

**Table 5:** Listing of the field names for the nomenclature of the file names.

Field	Explanation	Comment
Region	Region of the data set	e.g., Petermann Glacier
3D Horizon	3D Horizon abbreviation as shown in Table 3; e.g., P1 or P2 for the Petermann horizons	Note that the numbering does not represent the age chronology.
Version	Data set version (first version starting with v001)	e.g., if the dating of the age of the same horizon changes or if the data set is expanded
Age	Age of the data set in ka (thousand years with respect to the age of the ice core time scales). Note that the age is composed of two numbers (thousand years and a hundred years) separated by a “_”, where 12_0_ka represents 12.0 ka.	Here we do not include the age uncertainty
Subset (optional)	Capital letter (A, B, C,…) if one and the same age horizon is divided into different segments. This can occur when certain areas in a horizon were picked or interpolated with different accuracy.	
Geometry	Line or mesh	
File type	File ending	.ts and.pl for GoCAD objects,.tif and.nc for the gridded DEMs, and.dat for the xyz point ASCII data

### File structure in PANGAEA

The various data formats of the 3D Horizons are archived in a Pangaea Publication Series. The entire data collection has an overarching DOI^26^. Each 3D horizon is stored as a data set in Publication Series: FINEGIS F1, FINEGIS F2, FINEGIS F3, NEGIS N1, Petermann P1, Petermann P2, Northern Central Greenland NG1, Northern Central Greenland NG2 (Table 6). Each data set contains several files of different data formats (ASCII, DEMs, GoCAD). Each dataset (e.g., FINEGIS F1) in turn, has its own DOI (Table 6). The download options can be accessed via the “View dataset as HTML” section. The entire dataset (i.e., all file formats) can be downloaded as a.zip or.tar archive, or the individual file formats can be downloaded separately.

**Table 6:** Listing of the data sets published in this manuscript via the Pangaea Publication Series.

Data Set	Title	DOI
FINEGIS F1	Three-dimensional stratigraphic horizon in the northern upstream region of 79 NG, Greenland ice sheet (45.5 ka).	10.1594/PANGAEA.954895
FINEGIS F2	Three-dimensional stratigraphic horizon in the northern upstream region of 79 NG, Greenland ice sheet (52.2 ka).	10.1594/PANGAEA.955046
FINEGIS F3	Three-dimensional stratigraphic horizon in the northern upstream region of 79 NG, Greenland ice sheet (60.0 ka).	10.1594/PANGAEA.955103
NEGIS N1	Three-dimensional stratigraphic horizon in the upstream region of the Northeast Greenland Ice Stream, Greenland ice sheet (7.3 ka)	10.1594/PANGAEA.955104
NC Greenland NG1	Three-dimensional stratigraphic horizon in the Northern Central Greenland ice sheet (13.8 ka)	10.1594/PANGAEA.955183
NC Greenland NG2	Three-dimensional stratigraphic horizon in the Northern Central Greenland ice sheet (37.5 ka)	10.1594/PANGAEA.955191
Petermann P1	Three-dimensional stratigraphic horizon in the Petermann Glacier region, Greenland ice sheet (12.0 ka)	10.1594/PANGAEA.955194
Petermann P2	Three-dimensional stratigraphic horizon in the Petermann Glacier region, Greenland ice sheet (37.5 ka)	10.1594/PANGAEA.955196

The entire Publication Series has the following DOI: 10.1594/PANGAEA.954991^26^.

## Technical Validation

### 3D horizon geometry validation

**Fig. 6:**
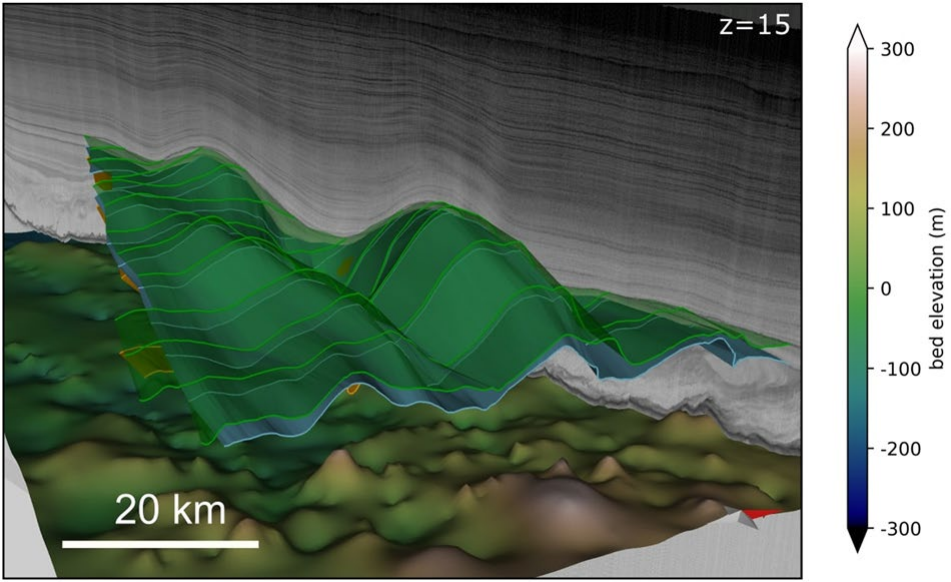
Radargram that is oblique to the traced IRHs used for the 3D horizon construction and can be used to validate the resulting 3D horizon geometry independently.

For the 3D horizon construction, we preferred to use parallel RES profiles. For validating the interpolated geometries, we use those RES profiles that run obliquely to the main ones that were not used in the 3D horizon construction (Fig. 6). We have carried out this validation of the 3D geometry for all horizons published here.

### Age validation

Because we converted our time-domain RES data into the elevation-domain with a constant dielectric permittivity of ε_ice_ = 3.15, we also applied a firn correction of 10 m^81^ in all our horizons when linking the depth of the isochrone with the depth of the ice core. Furthermore, we considered various factors that may introduce a potential age error, such as (1) the range resolution of the RES system of ∼4.31 m^58,71^; (2) uncertainties in the determination of the surface reflection in the RES data (estimated uncertainty up to ∼5 m); (3) an error due to inaccuracies by the user who traces the isochrones in the radargrams (estimated to be two times the RES system range resolution; ∼10 m); (4) an error in the respective age scale (<1 m); (5) spatial variations in the density-depth function, either manifesting in a slightly different permittivity (e.g., from anisotropy) or a different firn correction (estimated to ∼5 m). Overall, we estimated the uncertainty in the age assignment to the horizons to be approximately ± 25 m relative to the depth of the respective ice-core time/depth scale. This means that the overall age error increases with depth (Table 3). Normalized to the absolute ages, the estimated age errors range between 2.5 and 7.5%. The errors given for the dating of the 3D horizons are thus relatively large, but we see potential in the future to minimize the errors for upcoming and existing 3D horizons by performing more precise determinations of the IRH ages, for example, with the use dielectric profiling measurements in combination with synthetic radar modelling^78^.

## Usage Notes

### Summary of data records

#### Petermann glacier

**Fig. 7:**
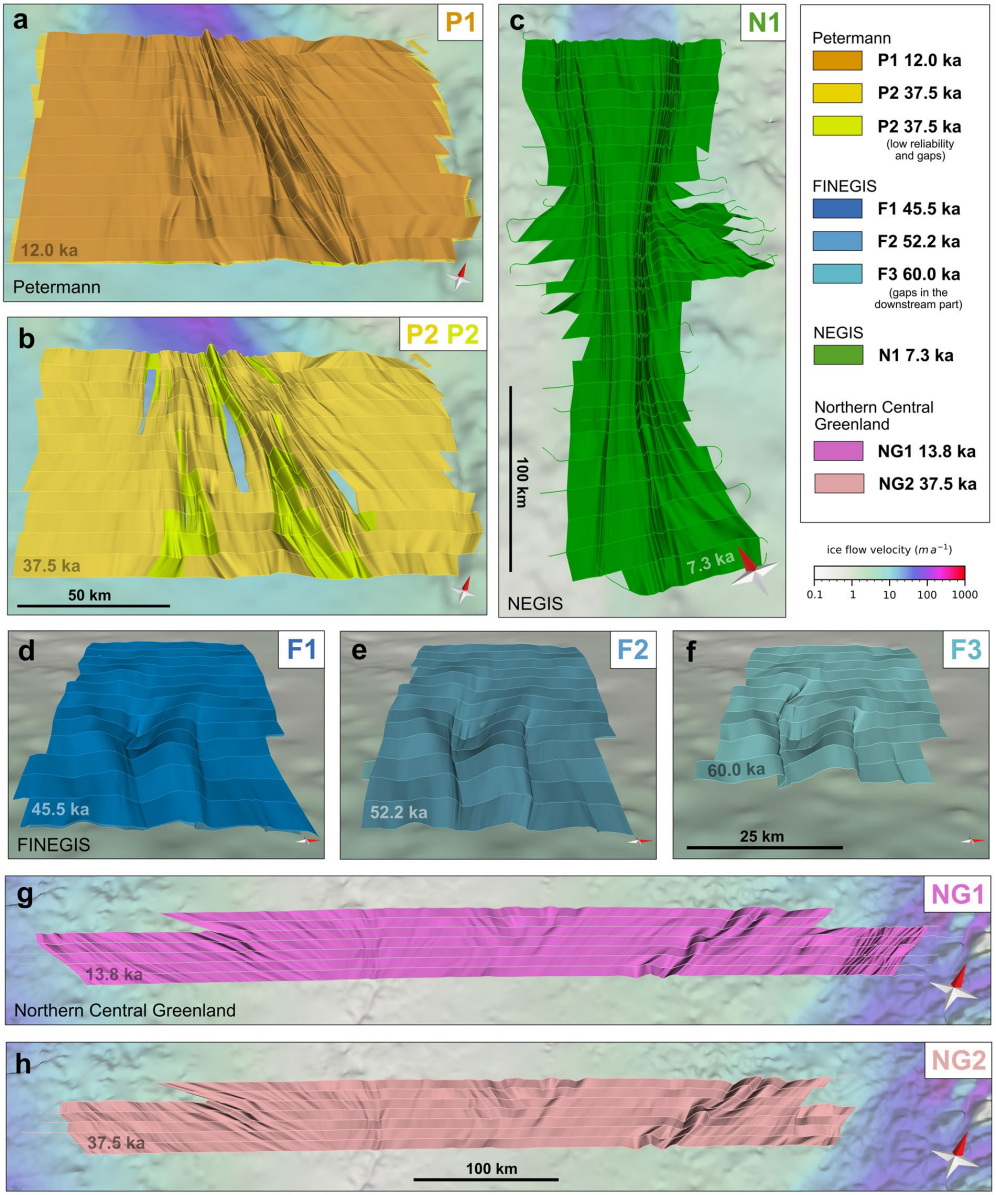
Individual 3D horizons published with this manuscript. Panels (**a,b**) represent the horizons from the onset of the Peterman Glacier, (**c**) the horizon at the NEGIS onset, centred at the EGRIP drill site, (**d**–**f**) the horizons upstream of the northern catchment of the 79NG (FINEGIS) and (**g,h**) the horizons covering northern central Greenland. The map in the background shows the bed topography^47^ overlain with a colour map of the surface ice flow velocity of Greenland^85^. It should be noted that the 3D horizons in are vertically exaggerated along the elevation axis by a factor of 15 for better visualization. A comparison between a vertically exaggerated 3D horizon and a non-exaggerated one representing the actual scale ratios is shown in Fig. 8 for the Petermann Glacier dataset.

The data set from the onset region of Petermann Glacier^19^ consists of two 3D horizons (P1 and P2), which cover an area of approximately 156 × 95 km. Horizon P1 is ∼12.0 ± 0.9 ka old and approximately represents the transition from the Last Glacial period to the Holocene period and is located on average 1 km below the ice surface. The horizon is mainly characterized by open cylindrical folds with the fold axis oriented parallel to ice flow^19^ (Fig. 7a). The amplitudes of the folds reach up to 1 km with a wavelength of 10–15 km and are highest in the centre of the data set, corresponding to the region where ice flow converges towards the outlet glacier downstream. The deeper horizon P2 is ∼37.5 ± 2.1 ka old and shows increased folding intensity with slightly asymmetric and overturned folds. Nevertheless, the folds in P2 mimic those of the shallower P1 horizon (Fig. 7b). In contrast to P1, P2 shows gaps in regions where the 3D horizons could not be created due to uncertainties or poor visibility of the IRHs. Therefore, the datasets provided for horizon P2 are subdivided into P2A and P2B.

#### Northeast greenland (FINEGIS)

The data set in northern Greenland upstream of the northern branch of the 79NG represents three 3D horizons (F1, F2 and F3), which are located close to the ice divide (Fig. 1). On average, the data span an area of 105 × 65 km. All three horizons show two cylindrical fold units that are visible in the lower third of the ice column^21^. The fold axes of these folds trend towards 100° (relative to true north) and show an increase in the degree of deformation (larger fold amplitudes) from the upper to the lower horizons (Fig. 7d–f). In contrast to the Peterman fold axes, the fold axes are oblique (∼25°) to the surface flow direction^21^. F1, F2 and F3 have ages of 45.5 ± 2.5, 52.2 ± 2.7, and 60.0 ± 4.0 ka, respectively. The two cylindrical folds are systematically overturned towards the north, whereby the degree of overturning increases downwards from F1 to F3. The deepest and oldest horizon, F3, is about 10% smaller in surface area than the two shallower horizons (Table 3).

#### NEGIS onset

The data set at the onset of NEGIS consists of one single 3D horizon (N1; 7.3 ± 0.2 ka), which covers an area of ∼250 × 90 km and is centred on the EastGRIP drill site (Fig. 1). The geometry of this 3D horizon shows a variety of complex folds with increasing fold density and intensity in the downstream direction. The shear margins are characterized by small wavelength (100–500 m) and small to moderate amplitude (∼100 m) folds that are oriented approximately parallel to the shear margins^58^. Each of these folds can be traced for tens of kilometres. In addition, long-wavelength (1–10 km) cylindrical folds are observed outside the ice stream. These folds form a fan-like pattern extending out from NEGIS (Fig. 7c). Close to the ice stream, the folds rotate towards parallelism with the shear margins while their wavelength decreases at the same time.

#### Northern central greenland

The data set in northern central Greenland covers the largest region in this data collection and consists of two horizons. The 3D horizons cover an area of approximately 690 × 55 km, reaching almost from the eastern to the western ice-sheet margins (Fig. 1). The shallower and thus younger horizon (NG1; 13.8 ± 1.0 ka old) is slightly larger in area than the deeper and older horizon (NG2; 37.5 ± 2.1 ka old). NG1 extends in the east into the NEGIS trunk and shows short-wavelength folds similar to those observed in the NEGIS shear margins (horizon N1; Fig. 7g). NG1 and NG2 reveal several upright cylindrical folds at each end, i.e., in the east and west of the covered area. The axes of the cylindrical folds in the west trend approximately west, whereas in the east, they trend towards the north. In the central-western area of NG1 and NG2, we find a large trough in the bed topography due to the paleofluvial mega-canyon^65^, which is imprinted in the 3D horizons that mimic this bed depression. In the centre of the covered area, near the main ice divide, we find upright cylindrical folds that trend to the north and northwest (Fig. 7g,h).

### Potential applications of 3D horizons

The utilization of a 3D representation of IRHs in Greenland and Antarctica can contribute significantly to future studies and expand our understanding of the present-day and past behaviour of these environments. The features visible in the single sections are not necessarily linked directly to bed topography and surface ice flow velocity, data which is often used to plan the grid of a radar survey. The added value of a 3D horizon in comparison to the analysis of single IRHs is that it immediately reveals the geometry of structures independently of the grid layout. By examining the depth, continuity and geometry of these 3D horizons we can infer information on e.g., past and present ice flow patterns, basal conditions and properties and internal deformation processes^19,21^. Future studies can use this three-dimensional information to calibrate and refine ice-sheet models^82^,^83^ assess ice mass loss or gain and better predict the response of both ice-sheets to a warming climate. 3D horizons also provide a detailed view of the age structure within an ice sheet and provide, thus, valuable information on ice-sheet structure and dynamics. Furthermore, 3D horizons hold the potential to reveal information about past accumulation^23,84^. Altogether, this knowledge is crucial for understanding ice-sheet stability and their influence on ice-sheet dynamics .

**Fig. 8:**
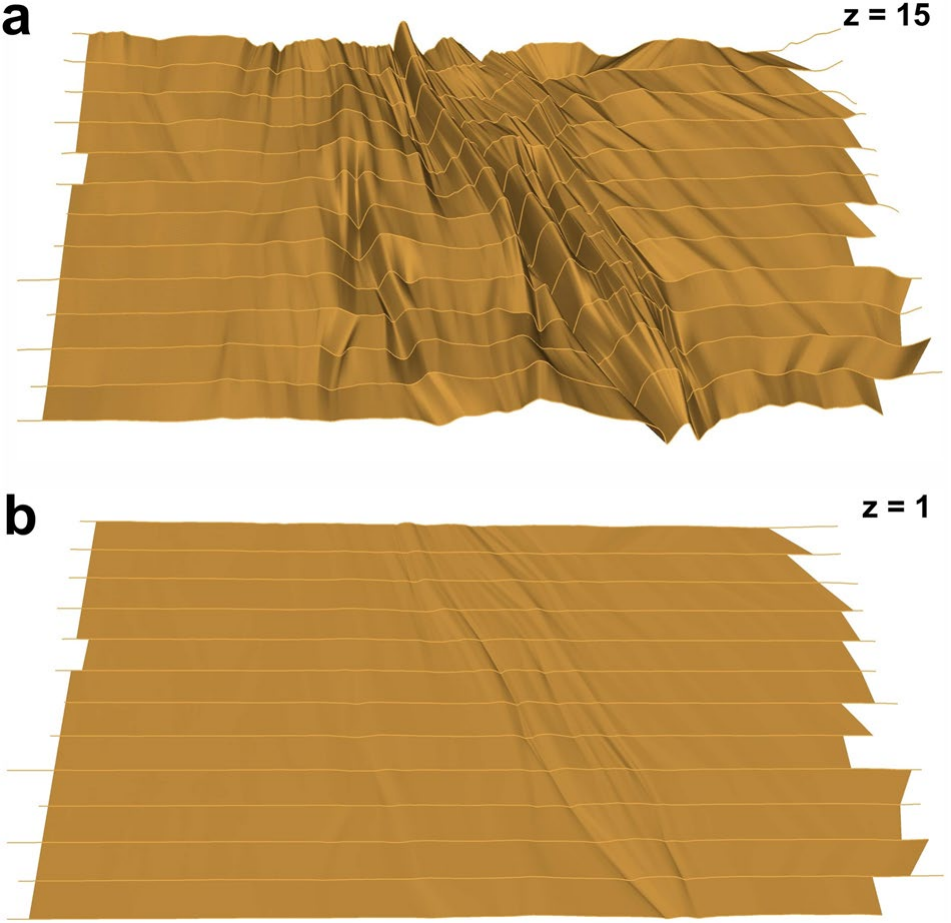
Difference between a fifteen-times vertically exaggerated (**a**) and not exaggerated (**b**) view on the Petermann Glacier P1 12.0 ka horizon.

## Data Availability

The CReSIS toolbox used to process the MCoRDS RES data is available at https://gitlab.com/openpolarradar/opr, and the main documentation can be found at https://gitlab.com/openpolarradar/opr/-/wikis/home.
